# A phenomenological understanding of residents’ emotional distress of living in an environmental justice community

**DOI:** 10.1080/17482631.2016.1269450

**Published:** 2017-01-05

**Authors:** Gabriela Dory, Zeyuan Qiu, Christina M. Qiu, Mei R. Fu, Caitlin E. Ryan

**Affiliations:** ^a^ US Army Armament Research, Development and Engineering Center, Picatinny, USA; ^b^ Department of Chemistry and Environmental Science, New Jersey Institute of Technology, Newark, USA; ^c^ Harvard College, Harvard University, Cambridge, USA; ^d^ College of Nursing, New York University, New York, USA; ^e^ North Shore University Hospital Manhasset, New York, USA

**Keywords:** Emotional distress, environmental justice, qualitative research, descriptive phenomenology, Ironbound, Newark

## Abstract

Deteriorative environmental conditions in environmental justice (EJ) communities not only post direct health risks such as chronic illnesses, but also cause emotional distress such as anxiety, fear, and anger among residents, which may further exacerbate health risks. This study applies a descriptive phenomenological method to explore and describe the emotional experience of residents living in Ironbound, a known EJ community located in Newark, New Jersey. Twenty-three residents participated in the study. Four essential themes regarding the residents’ emotional experiences were elicited from 43 interviews: (1) being worried about the harmful effects of the surrounding pollution; (2) being distressed by the known historical pollution sources; (3) being frustrated by the unheard voices and/or lack of responses; and (4) being angered by the ongoing pollution sources. Participants not only expressed their emotions of worry, distress, frustration, and anger in detail but also described reasons or situations that provoked such negative emotions. Such detailed depictions provide insights into potential meaningful strategies to improve residents’ psychological wellbeing by alleviating negative emotions and meaningfully engaging residents in developing, implementing, and enforcing environmental laws, regulations, and policies to achieve EJ goals.

## Introduction

Environmental justice (EJ) is defined as the fair treatment and meaningful involvement of people of all racial, educational, economic, and social backgrounds in the development, implementation, and enforcement of environmental laws, regulations, and policies and aims to ensure everyone enjoys the same degree of protection from environmental and health hazards as well as equal access to the decision-making process to have a healthy environment in which to live, learn, and work (US Environmental Protection Agency [EPA], 2011). Environmental justice specifically addresses situations where minority or low-income communities bear disproportionately high and adverse human health or environmental risks. Such communities where residents have disadvantaged social, educational, and economic backgrounds are referred to as communities of concerns, or EJ communities (EPA, 2015).

Despite significant efforts for achieving EJ in these communities, continuous environmental problems persist where residents are still exposed to environmental hazards on a daily basis (Buzzelli, Jerrett, Burnett, & Finklestein, 2003; Hipp & Lakon, 2010; Mitchell & Norman, 2012; Walker, 2010). Such exposure becomes widespread in some instances as some EJ communities have experienced population growth as a result of lower housing prices (Baden and Coursey, 2002). Physical exposure to environmental hazards causes physical health problems, such as cancer, asthma, and other chronic illnesses (Collins, Grineski, Chakraborty, & McDonald, 2011; Corburn, 2007; Gilbert & Chakraborty, 2011; Hoek, Brunekreef, Goldbohm, Fischer, & van den Brandt, 2002; McConnell et al., 2006). Perceived environmental hazards also impact individuals’ psychological wellbeing by eliciting anxiety, fear, and stress (Böhm, 2003; Bullinger, 1989; Elliott, Cole, Krueger, Voorberg, & Wakefield, 1999; Marques & Lima, 2011). Negative emotions may further exacerbate health risks and induce chronic illness (Choi, Rush, & Henry, 2013; Franks et al., 2012; Kiecolt-Glaser, McGuire, Robles, & Glaser, 2002). Qualitative studies using the method of drawing and interviews shows that residents living in EJ communities are acutely aware of the waste facilities in their neighborhood and that they perceive waste facilities as a bad presence in their lives, which evokes negative emotions of fear, stress, and feelings of injustice (Lejano & Stokols, 2010; Pluhar, Piko, Kovacs, & Uzzoli, 2009). In such studies, the concepts of anxiety, fear, and stress were pre-assumed to assess psychological effects of pollution (Bullinger, 1989; Marques & Lima, 2011). Adopting pre-assumed concepts of anxiety, fear, and stress is to decontextualize individuals’ daily interactions with a broader context of living in an EJ community.

The purpose of this study was to explore and describe the emotional experiences of individuals living in an EJ community using a descriptive phenomenology method. Descriptive phenomenology follows the tradition of Husserl (1962) to explore individuals’ experiences of everyday life, describe the structure of such experiences and provide a thorough understanding of shared experiences (Sokolowski, 2000; van Manen, 2014). A descriptive phenomenological method allows the exploration of individuals’ emotional experiences of living in an EJ community without any pre-assumptions in order to reveal how individuals respond emotionally to their daily experiences of living in an EJ community and how such emotional experiences influence their daily lives. Such exploration can expand our understanding of living in an EJ community beyond the concepts of anxiety, fear, stress, feelings of injustice, and conflicting or negative feelings. A thorough understanding of emotional experiences of individuals living in an EJ community would be the first step to meaningfully engage individuals in the communities in developing, implementing, and enforcing effective environmental laws, regulations, and policies to achieve EJ goals.

## Data and methods

### Descriptive phenomenological method

The relationship between people and environment has been the subject of extensive phenomenological inquiries. Such inquiries include place, place attachment, and place identity (Casey, 2009; Donohoe, 2014; Malpas, 2007; Mugerauer, 1994; Relph, 1976; Seamon, 2014; Stefanovic, 2000). Although a phenomenological method offers an important perspective to understand environmental justice (Seamon, 2013), it is rarely applied to describe and understand the residents’ lived experiences in EJ communities, with few notable exceptions. (Ceaser, 2015; Lejano & Stokols, 2010). In this study, a descriptive phenomenological method with a qualitative and cross-sectional design (Fu & Rosedale, 2009; Fu, Xu, Liu, & Haber, 2008; Porter, 1998) was employed to ensure a deep understanding of the uniqueness of each participant’s emotional experience and the shared experience of all participants, that is, the essence or common patterns or universality of the experience (Husserl, 1962). The philosophical underpinnings of the study were based on the essential beliefs of Husserlian descriptive phenomenology from which certain assumptions are grounded (Husserl, 1962). According to Husserl, “natural knowledge begins with experience (*Erfahrung*) and remains within experience” (p. 45). Therefore, the individuals’ emotional experiences of living in an EJ community must emerge from the experience in which individuals interact with the living conditions of the community. Husserl believes that “every experience…has intentionality” (p. 222). This study assumes that individuals who live in an EJ community are able to purposefully reflect on their experience of interacting with their living conditions. Because the essence of individuals’ experience, that is, “essential universality” (p. 47) or “essential generality” (p. 53), “can be exemplified intuitively in the data of experience” (p. 50), the essence of the emotional experience of living in an EJ community can be achieved by depicting the perceptions and reflections of individuals who share their experiences of living in an EJ community.

To ensure the phenomenon under study is described as it is, without bias and preconceptions, a phenomenological reduction was conducted to bracket conventional knowledge about the phenomenon prior to data collection. The process of “bracketing” was achieved through formal sessions where researchers discussed the existing literature and personal understandings of the experience (Denzin, 1989; Fu et al., 2008; Porter, 1998). Two key ideas representing conventional knowledge were bracketed: (1) anxiety, fear, and stress are major psychological effects of pollution (Bullinger, 1989; Marques & Lima, 2011); and (2) individuals’ perceptions of an EJ community may elicit fear, feelings of injustice, and conflicting or negative feelings (Atari, Luginaah, & Baxter, 2011; Lejano & Stokols, 2010; Pluhar et al., 2009).

### Study site

The study was conducted in Ironbound, a multi-ethnic, working class community located in the East Ward district of Newark, Essex County, New Jersey. Ironbound acquired its name from the railroad tracks that once surrounded the area on three sides. Highways, including Routes 1 and 9, 21, 78, and the New Jersey Turnpike, and Liberty Newark International Airport bound Ironbound to the South, the Passaic River on the north, Penn Station and the Amtrak Line on the west, and Ports Newark and Elizabeth on the East. Ironbound has about 50,000 residents, and two-thirds of them are immigrants, largely from Central and South America. The US Environmental Protection Agency (2010) identified Ironbound as an EJ community with disproportionately high levels of environmental hazards and a low-income immigrant population. Ironbound has multiple pollution sources, including the municipal solid waste incinerator, daily heavy motor vehicle traffic, daily heavy airplane traffic from Liberty Newark International Airport, daily heavy sea vessel traffic from Elizabeth Port Authority Marine Terminal and Port Newark, railroad cars, and soil and water contamination in the Passaic River and Newark Bay accumulated from past and current industrial operations.

### Data collection

A semi-instructed interview was used to explore and describe the emotional experiences of residents living in Ironbound. The interview questions were carefully conducted to avoid introducing any bias and pre-assumption regarding their emotional experiences. Instead of directly asking the participants to describe their emotional experience of anxiety, fear, stress, feelings of injustice, or negative feelings, each participant was asked to answer a broader question: “What is it like for you to live in your community?” as well as specific questions: (1) “Please tell me what you like most about your community?” (2) “Please tell me what concerns you about your community?” and (3) “Please describe what feelings you have about your community.” General probes were also used to elicit more detailed information, such as “Please tell me more about that” “How did that make you feel?” and “What else did you do?” The New Jersey Institute of Technology Institutional Review Board (IRB) reviewed and approved the interview guide, including the questionnaire. Informed consent was secured from all participants. Privacy was ensured in that all the interviews were conducted in private settings. A coding system with numbers replacing participants’ names was used to ensure confidentiality.

A purposive sampling technique was employed (Fu & Rosedale, 2009; Fu et al., 2008) to recruit residents who lived in the community for the study by starting with a few individuals in the community who the researchers knew. Additional participants were recruited by following the recommendations of these individuals who participated in the study. The inclusion criteria were: (1) being 21 years of age or older; (2) having been a resident of Ironbound for at least two years before enrolling in the study; (3) being able to communicate in English; and (4) living within two miles of the municipal solid waste incinerator.

The sample size for a descriptive phenomenological study is determined by the richness and saturation of the data, that is, when the same information has been repeated by the participants from each other regarding the description of their experiences (Fu & Rosedale, 2009; Fu et al., 2008; Morse, 1994). Strong convergence emerged when interviewing the twenty-first participant. To ensure that all important information was captured, two extra participants were enrolled and interviewed. Data saturation was assured when no more new information emerged in the interviews of the last two participants. Altogether, 23 participants were interviewed. Of the 23 participants, 13 were female and 10 male. The average age of the participants was 46.6 years old, with the youngest being 21 and oldest 70. On average, participants had lived in the community for 28.7 years, with a minimum of 3 and maximum of 69 years. Eight participants had a White-Caucasian cultural background, two Portuguese, five Brazilian, seven Spanish, and one French. Five participants had a masters degree, 12 a bachelors degree and six below bachelors. Three participants were retired and one was a university student, the remaining participants had a variety of careers: legislator, teacher, artist, paralegal, nanny, restaurant owner, retailer, librarian, bookkeeper, seaport operator, architect, and cleaning worker.

Participant recruitment and interviews occurred between February and November 2013. Participants were interviewed twice to ensure consistency and a complete description of their experiences. In-depth interviews were conducted using the interview guide approved by the New Jersey Institute of Technology IRB. A second interview was conducted based on the same interview guide within two months of the first interview. One participant was unavailable for a second interview and two other participants were dropped from the study. In total there were 43 interviews including 23 first interviews and 20 second interviews. Each interview lasted from 70 to 140 min and was recorded using a digital audio-system, observational data for each participant were also recorded. All the interviews were professionally transcribed and checked for accuracy. The quality of interviews and transcriptions was ensured by checking each transcribed interview for accuracy. Data reliability was evidenced by the emergence of similar information elicited by the participants’ answers to the same questions during two interviews (Fu & Rosedale, 2009; Morse, 1994; Porter, 1998).

### Data analysis

The interview data were analyzed using a descriptive data analysis method based on intuitive reflections and strategies of continuously “comparing and distinguishing, collecting and counting, presupposing and inferring” (Husserl, 1962, p. 93). Crucial to this method is a systematic classification process of text data into fewer content-related themes that share the same meaning. We followed the seven-step data analysis procedure in Fu et al. (2008) to examine data, compare codes, challenge interpretations, and inductively develop themes. The steps were: (1) individually reading the transcripts several times to gain a broad understanding of the text; (2) meeting as a group to identify key quotations and discuss key codes related to the research question; (3) combining the coded quotations into one file and confirming the accuracy of the code and quotation; (4) carefully and individually analyzing quotation files and identifying major themes by putting key coded quotations together for each research question; (5) meeting as a group to review major themes together and engaging in active dialogue to resolve any discrepancies; (6) reviewing the transcripts and validating the structure of themes alongside interview data; and (7) conducting multiple discussions until consensus was achieved about each aspect of the process of data analysis. Efforts were made to differentiate and compare each individual’s experience with careful selection of text demonstrating the essence of the experience (Husserl, 1962; Porter, 1998). Credibility of data analysis was ensured by conducting numerous discussions until consensus was achieved about each aspect of the process. The essence of individuals’ emotional experience of living in an EJ community was fashioned into essential themes illuminating the meanings of the experience.

## Findings

Participants used the phrase “it is my home” to affectionately describe the community. Participants described Ironbound as a place where immigrants came because they could live close to their relatives, “That’s what the people who came over…emigrated over here did…the families kind of lived in clusters down in these areas.” Participants also were attracted to “the convenient location” of the community: “Everything is in walking distance,” “Being able to go down to Penn Station and get a train into New York City…makes it very important.”

Participants never labeled their community as “an EJ community,” yet they described the community as being “stigmatized and “unfairly burdened with a lot of industrial hazards” possibly because “it’s an immigrant community.” Participants articulated their concerns about “air pollution” caused by “exhaust from vehicles,” “sea vessels,” “airplanes,” “industry,” “the railroad,” and “the incinerator.” Participants were concerned about “water pollution” and “contaminated flood water” due to “the condition of the Passaic River.” Participants were also worried about “soil contamination” caused by historical industries and “houses built on contaminated land.” Participants expressed great worries about “noise” and “light” pollution generated by “the arena,” “heavy motor vehicle traffic,” ”exhaust from planes,” and the “pollution everywhere.”

It is within the paradoxical context of feeling the community was “home” and being concerned about the “heavily polluted community” that emerged the essential themes of their emotional experience of living in an EJ community.

### Being worried about the harmful effects of the surrounding pollution

Participants were concerned about the multiple environmental hazards in the community that included “noise,” “light,” “air,” “soil,” and “water” pollution. As one participant stated, “between the traffic congestion and then the trucks, we got the airport too, and all the factories, and then the incinerator.” Participants were very worried about the “horrible” harmful effects of pollution that had affected or could affect their health, such as “cancers,” “hearing loss,” “sleep disturbance,” “allergies,” “asthma and other respiratory problems,” and “autism.”

The health impact of noise pollution, such as “hearing loss,” “sleep disturbance,” and “autistic kids,” worried the participants enormously. As one participant stated, “I have earplugs [for] the noise pollution from the trucks. My hearing loss is contributed by the trucks and sirens you hear on Market Street. It’s really difficult to avoid all these noises.” One participant said, “the noise. It bothers me a lot.” Another said, “Noise pollution, air pollution, shakes the house and wakes me up when I’m trying to sleep.” Parents worried about the “health” impact from noise on their children. “Sleep disturbance” was another important worry for the participants. As one participant said, “I have to sleep with earplugs” and without them “I wouldn’t sleep.” Participants expressed worry about “light pollution” from “the flashing electronic signs and traffic.” As one participant described, “I cannot sleep. It’s really bad, the screen at the arena [the Prudential Center], it’s really bad, because, the light is consistently blinking, so even when you’re sleeping you’re getting affected by that, so it’s very disruptive.”

Participants were worried about the “seemingly pervasive cancers” in the community. Many participants either had personal experience of cancer or witnessed a friend or family member who had cancer. One participant, described a friend whose family lived adjacent to a factory and all died of cancer:

Directly behind her house is a company called Arol, A-R-O-L, and it’s on Ferry and Foundry…it’s a chemical company that’s been there since I was a child. And you’re gonna say, tell me, it’s just a coincidence that all these people died in that house of cancer? She buried every one of them from with cancer.

Another participant described how her pets got sick:

My cats, both of them got breast cancer they eventually died too, they couldn’t operate on it. It was spread. It’s sad to think of it. And I swear it’s because they are low to the ground. And if they are finding dioxin in your vacuum bag, my poor little kitty. My cats were indoors. I swear it [cancer] was from that [the pollution].

For the participants, the “smell” of the community was “the hallmark of air pollution” that “you cannot even pinpoint where the smell originated because it is so pervasive throughout all parts of Ironbound.” As one participant said, “the air just smells different” in Newark. One participant who grew up in a house under the path of the airplanes said that as a child he “spent a lot of time inside because of the smell that was outside.”

Participants attributed health problems of “allergies,” “asthma and other respiratory problems” to the air pollution in the community. One participant, a teacher in the local elementary school, commented, “In school we notice that a lot of the kids have asthma. And I’m like how do all these kids have asthma and it could be because of the incinerator, all the air pollution, all the toxins in the air.” One participant commented that she developed allergies when she moved to Ironbound: “Well in Brazil I didn’t have anything. Here, I have allergies. In the winter, I always have allergies and in the spring. Coughing a lot, I sneeze a lot. I wouldn’t sneeze at all in Brazil.” One participant with asthma said, “I avoid Ferry Street…there’s just too much exhaust.” Another participant said “it stinks when we’re driving, like it hits you. I don’t like the smell that, or breath in that…just have that dirty, dirty air.”

Being worried about the harmful effects of pollution on children was a paramount concern among the participants. Participants worried about the increased incidence of “autistic kids,” as one participant remarked: “We have a lot of kids who are autistic, a lot.” Participants speculated that autism was linked to the environmental condition, “I think all the immigrants in Ironbound know that the incinerator is causing heavy metal poisoning amongst them and their children.”

### Being distressed by the known historical pollution sources

Participants were very distressed by “the known historical pollution sources” in the community as they listed “the Passaic River,” and “contaminated land.” The participants portrayed the current condition of the community “being disgusting and disappointing” because of “lack of action” or “ineffective actions” to address the historical pollution sources. There was a feeling that residents were “being taken advantage of” by regulations that allowed polluted land to be “capped” or covered instead of remediated.

Historical industrial contamination was a contentious issue that elicited strong negative emotions among the participants. Participants were aware that “the main contamination from historical industries is contaminated land in Ironbound.” One participant said:

I did a little research and I went online and…[saw] all the different sites around that are actually polluting the ground so therefore the water…to find out, to be aware that just for the city of Newark there is over 200. There was a map that came up, was this section of New Jersey and okay, Livingston was in nice little yellow, some others were in blue, and Newark was a big fat red spot on it. Was I aware of it? Maybe I’ve been aware of it at some point, but I chose to ignore it. And I went back and looked at it and I’m, like, holy crap. So it was bad.

One participant commented on the new houses built on contaminated land: “I wouldn’t want to live there because I don’t know if it seeps up and people can get cancer or sick or something like that, so, and it’s a shame because the houses are, they do look nice, they’re brand new…they were pricy for being here in Newark, but it’s so contaminated that they had to seal the ground.” Participants expressed concern about the contaminated land, “a lot of those old industrial sites, the soil is so contaminated…you don’t want people touching it, never mind trying to grow something on it. For the participants, the remediation of historical industrial sites was disappointing, many participants were suspicious about “whether the cleanup was done properly or to the extent that was necessary.” As one participant remarked, “It upsets me that people let, let things get that bad and, factories and business and its money over the safety or the health of the environment and the people.” One participant said, “It offends me, one that you’re exposed to it and two that the powers to be didn’t really give a shit. They didn’t do anything about it. In fact, they went out and built homes over there.”

Participants especially expressed concerns about “capping,” a common pollution control practice in Ironbound to contain the “contamination left behind by former manufacturing companies” contamination or hazardous substances in place and prevent possible exposure to the contaminated land while allowing the land above the cap to be used for other purposes. Participants described “the popular trend of capping” in places with high industrial soil contamination. One participant with an architectural background felt that “capping was an inadequate form of land remediation.” As she said:

All that I can really say is visually now, what they’ve done, their mediation of it, which was to cap it, and put three or four pathetic planters on it, it’s pretty pathetic…I don’t consider capping taking care of it properly, [they] pushed it under the rug, or put a concrete rug over it, and maybe someday, someone will deal with it, but now it hasn’t been really dealt with….I don’t think that they did it properly, it’s still there.

Another participant said, “there are serious environmental challenges because of the history” of the pollution in the community and “I’m not quite sure if we’re cleaning these properties up at the levels that they should be.”

The Passaic River and the ongoing pollution caused by polychlorinated biphenyls in the river elicited strong emotions from participants. When talking about the current condition of the Passaic River, participants expressed audible disgust and great disappointment. Participants were “scared” and expressed “fear” about the contamination in the “gross” river. One participant said “That’s horrible [Passaic River]. I don’t think the quality of the water…I would never go near the Passaic River. I look at it and it’s murky and dark and I’m just like, god only knows what’s in that water.” As one participant commented, “The River looks so filthy, disgusting, [and] dirty.” Another participant said, “forget about the River. You’d probably melt.” Participants were distressed by the fact that the Passaic River remained contaminated and that the “water doesn’t seem…good even for the fish.” One participant said:

It’s sad, it’s very sad, because it could be a beautiful place…[but] you can’t enjoy it, not at all. A lot of people love to fish here, especially my…husband loves to fish, and he’s never going to fish in that River, it’s dirty, I’m sure about that, it’s completely polluted.

### Being frustrated by the unheard voices and/or lack of responses

Participants expressed excessive frustration evoked by the “lack of laws, regulations, and policy enforcement” that “help[s] to improve our community.” Participants were frustrated that their concerns about environmental hazards were “never heard by the community leaders and those with the power to improve the conditions.” Participants felt that they have “been discriminated against because of our lack of political power.” For the participants, “being frustrated by the unheard voices and/or lack of responses” was exemplified through a variety of instances in which their efforts to interact with various governments and authorities regarding environmental concerns were “in vain.” Participants felt that “the city government was unapproachable,” as one teacher remarked:

I have stopped going to any town meeting or anything like that because you feel…I feel like my town, my city is…the people who are in charge, they don’t see the bigger picture that Newark can be such a better city and over time it has become really, really negative and draining.”

Another participant who had tried to call City Hall to complain said, “trying to call town hall is probably like trying to call the court in Newark. You just get nasty people…nobody cares.”

Participants were frustrated by “the heavy truck traffic and subsequent exhaust emission that could have been improved with law and policy enforcement.” Participants were especially angered by truck idling that elicited unnecessary “horrible” air and noise pollution. They felt that “the lack of enforcement of idling laws encouraged vehicle idling.” One participant remarked:

The trucks have the engines running all the time because the drivers want either the air conditioning or heating [in the truck]. I’m choking on the fumes, I’m always calling the police to get rid of the idling trucks but I’m always having [a] hard time to get the police to come to stop the trucks.

In response to pollution from exhaust, one participant said: “there’s nothing I can do. I feel like what can I do?” One participant, 68-year-old retired worker, echoed a similar remark:

The thing that angered me was that the police would not respond to the calls. I called several times to the police when the trucks were idling under my windows and they would not respond. Sometimes, they [the police] answered the phone but they talked about they had ticket quotas and they could have come down and given that ticket because the ticket quotas were done. The DEP [Department of Environmental Protection] has written regulations when they give tickets for idling and pollution, the community gets the money. So it seems to me that it would be a win-win situation. So if you do enough of this you would see how fast this would stop.

Participants were exasperated by the pervasiveness of illegal dumping and “the lack of enforcement, [be]cause people actually drive into the city and down by Magazine Street down past where the highway is, people actually come and they dump the garbage.” One participant said, “every time you clean it up, they just dump again. They’re like little thieves in the night; they just dump again.” One participant recounted witnessing illegal dumping by her employer:

A couple times I was working I used to see - they would dump stuff. I would say, “What are you doing here?” He’d say, “Oh nothing, just don’t say anything. Nobody caught us.” I said, “But I saw you do it. It’s illegal. You’re not supposed to be dumping in there.”

Facing “pervasive illegal dumping or littering,” participants felt “mad and angry!” Participants’ frustration was highlighted by “the insufficient” or nonexistent responses of the city government to “illegal dumping and littering.” One participant said, “…the Government they just close their eyes [to] the community. They don’t care about the community. They don’t care about the people.”

### Being enraged by ongoing pollution sources

Participants expressed anger about “unbelievable” ongoing pollution sources in the community. The strong emotion of anger was highlighted when participants recounted their “unfruitful actions to fight against pollution sources.” Participants questioned the location of the incinerator and felt it “should be replaced not in an urban area.” One participants said “if I were buying a home today, would I buy it in Newark? No.” The municipal solid waste incinerator is one of the largest ongoing pollution sources in the community. Participants repeatedly expressed frustration and “helplessness” that their concerns against the incinerator were unheard by their community leaders. One participant recalled, “We were fighting against it. There were tons of meetings. We even went to council meetings. And they wouldn’t let people talk.” The participants described local government as “corrupt” and felt that government “did not care about the community’s concerns on building another pollution source in Ironbound” despite the fact that people took different actions against the incinerator, such as “signing petitions,” “going to meetings,” or “protesting.” One participant, 70-year-old restaurant owner, described:

People start to consider [Ironbound] a black hole, just throw anything down it, people don’t count, it’s already polluted, it’s already there. And that’s what happens, that’s what you’re fighting against. And the fight to get people to change their minds about what happens…we are people and we live down here and we don’t want the pollution, put it in your back yard, but they don’t wanna do that, it’s the NIMBY [not in my back yard] syndrome and they look in Ironbound and throw it down here.

For the participants, the incinerator not only “exacerbated the poor air quality in Ironbound but also increased truck traffic transporting municipal waste to the incinerator,” “trucks disposing the ash waste,” and “other commercial traffic necessary to run the facility.” Participants were infuriated by the fact that their community had to bear the burden of increased air pollution by “burning garbage for New Jersey and other surrounding states.” One participant said, “people have been screaming about it [pollution from the incinerator] for 30 years. People have been screaming about it and haven’t been able to get anything accomplished.” Some participants were enraged by the fact that “the incinerator in Ironbound is one of two waste incinerators among the five municipal waste incinerators in New Jersey that does not have an updated emission control system with the ability to reduce hazardous air emissions.” Participants also expressed feeling stigmatized, as one longtime resident said: “I personally think they just put too much in Ironbound….Why always Ironbound? Why can’t they put it [the incinerator] in another ward?”

## Discussion

Emotion is a cognitive process in which humans make sense of what they experience in a given environment. Negative emotions, such as anxiety, fear, stress, feelings of injustice, and conflicting and negative feelings, have been reported among residents living in EJ communities (Atari et al., 2011; Bullinger, 1989; Lejano & Stokols, 2010; Marques & Lima, 2011; Pluhar et al., 2009). This study signifies an initial effort to explore individuals’ emotional experience of living in an EJ community without adopting preconceptions of anxiety, fear, stress, and feelings of injustice, but focusing on the broader context of daily lived experiences among residents in an urban EJ community. Different from previous research, participants in our study, repeatedly and in vivid detail, portrayed the paradoxical context of their living in the community: feeling the community as “my home” on one hand, while being concerned about the “heavily polluted community” on the other; describing the processes of evoking negative emotions, that is: the awareness of harmful effects of the surrounding pollution provoked residents’ daily worry; the awareness of the known historical pollution sources elicited distress; the unheard voices of and/or lack of responses to their concerns about community conditions created frustration; and the ongoing pollution sources, instilled anger.

Living in the “heavily polluted community,” participants daily encountered “noise,” “light,” “air,” “soil,” and “water” pollution and confronted a myriad of daily challenges, in their words, which brought them “worry about the harmful effects of the surrounding pollution.” Such descriptions from the participants’ perspective confirm the general recognition that negative emotions such as anxiety, fear, anger, and stress are significant part of the lived experiences of individuals who are living in an EJ community due to the actual and potential health concerns related to environmental hazards.

It is important to note that these participants not only expressed their “worry,” “frustration,” “anger,” and “distress” of living in an EJ community, but also described in detail the reasons or situations that provoked negative emotions in their daily lives. In addition to the “worry” about their health conditions due to historical and ongoing pollution in the community, the participants were most “frustrated” and “enraged” by “the unheard voices” and “non-response” to their concerns from various governments or “the lack of laws, regulations, and policy enforcement” that “help to improve our community.” “The City was unapproachable” despite their numerous phone calls to the City Hall or police regarding “truck idling,” “illegal dumping,” “no-picking-up of garbage,” or “littering.” The municipal solid waste incinerator operates with outdated emission control technologies despite numerous protests and petitions. Such detailed depictions, from the participants’ perspective, have shed light on and provided insight into potential strategies that are meaningful to the improvement of participants’ emotional experiences and quality of life in EJ communities. For example, local government could take actions to install enough trash cans to prevent littering, to ensure timely response and enforcement of truck idling laws and illegal dumping and timely garbage collection, such actions would help to ease the negative emotions of frustration and anger among individuals living in an EJ community. A well designed website that provides opportunity for residents’ voices to be heard and posts useful information could help mitigate negative emotions. The residents’ anger and enragement about the air pollution related to the incinerator would be eased by updating its emission control system for the incinerator, which is a strict enforcement of the existing Clean Air Act regulation. Environmental justice is generally considered to the responsibility of the federal government, but the results of this study showed that the local government plays an extremely important role in improving the quality of life, especially reducing the emotional distress of residents living in EJ communities. Future EJ policy and strategy should address involvement of governments at all levels in their daily operations (EPA, 2011).

Research demonstrated that residents in EJ communities were excluded or had limited impact on legal and policy decisions that affected them (Lejano & Smith, 2006; Pastor, Morello-Frosch, & Sadd, 2005; Payne-Sturges, Burke, Breysse, Diener-West, & Buckley, 2004). The participants in this study still echoed the similar negative emotion of being “frustrated” and “enraged” because of the sentiment of being “unheard” and “ignored” by the community leaders or local government. Many factors affect the improvement of EJ communities. It is difficult and complicated to address historical and large-scale pollution issues that result in the EJ label for a community, such as the air pollution in Ironbound. However, there are many opportunities to engage local residents to take action, resolving many small-scale issues such as truck idling, illegal dumping, and noise and light pollution, which could significantly improve residents’ emotional experiences of living in the community.

Despite these negative emotional experiences in their daily encounters with pollution sources in the community, the residents did describe instances when they were empowered through the shared experiences. The residents were organized primarily through local non-governmental community groups such as the Ironbound Community Corporation. One example was their collective effort to save the Riverbank Park from developing into a sports arena. Residents described feeling “involved” in their community and “strength in number[s]” in such actions. Residents who were successfully organized, regardless if they achieved their goals, described the benefits of “work[ing] with the community.” Residents felt their community was marginalized and it is subject to themselves to take action for improvement. As one resident said, “we are people and we live down here and we don’t want the pollution, put it in your back yard….And this is what the people fight against, which I fight against….” The shared emotional experiences described in this research provide powerful narrative to unify the residents in a disenfranchised community to conduct self-advocacy for inclusion into a political process that brings positive changes to the community (Butler & Adamowski, 2015).

## Conclusion

Attention to the emotional wellbeing, an important domain of quality of life, among residents living in EJ communities is of ultimate importance to improve quality of life in EJ communities. This study uniquely applied a descriptive phenomenological method studying an under-reported condition (emotional experience of residents) and under-studied perspective (their lived experiences of living in an EJ community), which may reflect people’s cognitive process of trying to make sense of living in EJ communities. The study results were based on 43 interviews with 23 residents in Ironbound. Through the relatively rich and in-depth data from the 43 interviews, the study examined closely the underlying reasons or situations that provoked negative emotions of living in an EJ community. Since each EJ community has its unique situation in terms of pollution, residents, or local policy, the findings of the study may not be generalized to represent the universal emotional experiences of living EJ communities. However, the findings from this study provide unique insight into the residents’ emotional experiences and shed light on potential strategies that are meaningful in improving the quality of life of the residents in an EJ community.
